# Influence of neurokinin B, dynorphin A and kisspeptin-10 on *in vitro* gonadotropin secretion by anterior pituitary cells isolated from pubescent ewes

**DOI:** 10.2478/jvetres-2025-0003

**Published:** 2025-01-31

**Authors:** Natalia Szysiak, Urszula Kosior-Korzecka, Vincenzo Longo, Krzysztof Patkowski, Monika Greguła-Kania, Aneta Nowakiewicz, Mariola Bochniarz, Andrzej Junkuszew

**Affiliations:** Sub-Department of Pathophysiology, Lublin, Poland; National Research Council, Institute of Agricultural Biology and Biotechnology, Research Unit of Pisa, 56124 Pisa, Italy; Department of Animal Breeding and Agricultural Advisory, Faculty of Animal Sciences and Bioeconomy, University of Life Sciences in Lublin, 20-950 Lublin, Poland; Sub-Department of Veterinary Microbiology, Department of Preclinical Veterinary Sciences, Faculty of Veterinary Medicine, Lublin, Poland

**Keywords:** neurokinin B, dynorphin A, kisspeptin, gonadotropin, puberty

## Abstract

**Introduction:**

The hypothalamic neuropeptides kisspeptin-10 (KiSS-10), neurokinin B (NKB), and dynorphin A (Dyn A) play roles in the endocrine regulation of the hypothalamic–pituitary–ovarian (HPO) axis in puberty. Livestock’s timely attainment of sexual maturity increases reproductive efficiency and raises profitability. The pituitary relationship between these neuropeptides and gonadotropins in puberty in ewes being undercharacterised. The aim of the study was to analyse their direct effect on gonadotropin secretion by pituitary cells isolated from pubescent ewes.

**Material and Methods:**

Cells were incubated in McCoy’s 5A medium, either without neuropeptides (as the control) or with 10^−11^, 10^−10^, 10^−9^, 10^−8^ and 10^−7^ M of KiSS-10, NKB and Dyn A. After 4, 12 and 24 h, the luteinising hormone (LH) and follicle-stimulating hormone (FSH) concentrations were analysed by ELISA using species-specific antibodies.

**Results:**

Greater LH and FSH secretion was observed after the 4–24 h exposure to respective 10^−11^–10^−8^ M and 10^−11^–10^−7^ M concentrations of KiSS-10. Moreover, NKB and Dyn A applied in the concentration range elevated the secretion of both LH and FSH throughout the experiment. Dynorphin A had the most significant effect on gonadotropin secretion at all the concentrations used. In contrast, the most pronounced dose-dependent neuropeptide effect throughout the experiment on the FSH secretion was attributed to NKB.

**Conclusion:**

Kisspeptin-10, NKB and Dyn A had a direct impact on gonadotropin secretion by ovine pituitary cells. However, a detailed explanation of their role in gonadotropin secretion by the anterior pituitary gland in sheep and of their impact on the regulation of the HPO axis during sexual maturation or in the pathomechanism of delayed puberty require further studies.

## Introduction

**Fig. 1.**
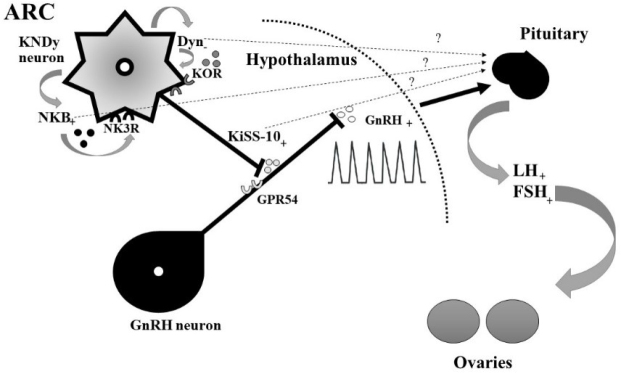
Role of kisspeptin-10 (KiSS-10), neurokinin B (NKB) and dynorphin (Dyn) A in the endocrine regulation of puberty, based on Navarro (20) and Pinilla *et al*. (24) ARC – arcuate nucleus; KNDy – kisspeptin, neurokinin B and dynorphin; GPR54 – G protein-related membrane receptor 54; NK3R – neurokinin 3 receptor; KOR – kappa opioid receptor; GnRH – gonadotropin-releasing hormone; LH – luteinising hormone; FSH – follicle-stimulating hormone; + – positive action; − – negative action; ? – unknown action

Puberty is a multifactorial and complex process in animal development resulting in the attainment of the reproductive capacity needed for species survival. It is known that there are many internal and external factors controlling it, among others achievement of appropriate age and weight, metabolic state, photoperiod, susceptibility to stress, and nutrition (32). In the case of livestock, timely attainment of sexual maturity contributes to increased reproductive efficiency, which leads to higher profitability. Any deviation in the maturation process has its negative consequences for proper development. The role of some neuronal factors as gatekeepers of puberty is not completely known and still under research. Many studies revealed that kisspeptin, neurokinin B and dynorphin neuropeptides, collectively referred to as KNDy neuropeptides and recognised as the key neuropeptides produced and secreted by the arcuate nucleus of the hypothalamus (ARC), are involved in the endocrine regulation of the onset of puberty (11, 20, 24) (Fig. 1).

Kisspeptins (KiSS), encoded by the *kiss-1* gene, are a group of endogenous ligands of orphan G protein-related membrane receptors (GPR54) (10). The first product of *kiss-1* gene expression is their common precursor, prepro-kisspeptin, and its proteolysis results in the formation of subsequent peptides, such as kisspeptin-54, kisspeptin-14, kisspeptin-13 and kisspeptin-10 (KiSS-10) belonging to the RF-amide peptide family (24). The KiSS/GPR54 system is a factor that controls the hypothalamic–pituitary–ovarian (HPO) axis. It is suggested that KiSS may play a crucial role in the emergence of reproductive function at puberty in both humans and animals. In sheep, the preoptic area kisspeptin population in the hypothalamus is thought to be involved in surge generation, and ARC kisspeptin neurons are engaged in the regulation of both surge and tonic gonadotropin-releasing hormone (GnRH) secretion in response to 17-beta-oestradiol (E2) (15, 24). Redmond *et al*. (28) reported that intravenous administration of KiSS in prepubertal ewes enhanced pulse-like and surge-like secretion of LH. Aerts *et al*. (1) demonstrated that injections of neurokinin-3 (NK3)-saporin into the ARC caused ablation of KNDy neurons in ewes and, consequently, a delay in puberty. Any abnormalities in the KiSS/GPR54 system may cause various forms of reproductive disorders, *e.g*. delayed puberty or idiopathic hypogonadotropic hypogonadism (5).

Neurokinin B (NKB), encoded by the *TAC3* gene in ewes (12), is a 10-amino-acid peptide belonging to the tachykinin family, which also includes neurokinin A, substance P and γ neuropeptides (24). Tachykinins have a common carboxy-terminal Phe-X-Gly-Leu-Met-NH2 amino-acid sequence, where X can be an aliphatic or aromatic amino acid (20). To date, three tachykinin receptors have been identified: for neurokinin-1 (NK1R), neurokinin-2 (NK2R) and neurokinin-3 (NK3R); the last one is a receptor for NKB (24). Neurokinin-3 receptor is principally found in the central nervous system, where it is co-expressed by practically all KNDy neurons in the ARC (22). The NKB/NK3R system is regulated by sex hormones and is suggested to be one of the main modulators of the hypothalamic–pituitary–gonadal axis (22). Acting locally on KNDy neurons, NKB stimulates the release of KiSS, which binds to its receptor on GnRH neurons, thereby increasing the secretion of GnRH (2). Neurokinin B is an important component of the mechanism responsible for the initiation of the ovine sexual maturation process (3). Nestor *et al*. (23) reported that single intravenous administration of senktide (an NKB agonist) increased LH secretion in prepubertal female sheep.

Dynorphin A (Dyn A), encoded by the *Pdyn* gene, is a compound from the family of endogenous opioid peptides, which also includes β-endorphin and enkephalin, and is characterised by the presence of a common N-terminal Tyr-Gly-Gly-Phe-Leu (or Met) amino-acid sequence (34). The receptor for dynorphin is the kappa opioid receptor (KOR), which is highly expressed in the central nervous system, especially in KNDy and GnRH neurons (37). Some reports indicated that Dyn A inhibited LH secretion in rodents (6, 20). Moreover, Dyn A may also be involved in the regulation of reproductive processes in ruminants. Lopez *et al*. (13) demonstrated that intracerebroventricular infusion of the KOR antagonist norbinaltorphimine notably enhanced mean LH levels compared to the control group in ovariectomised ewes at a prepubertal age treated with an oestrogen implant. There are also single findings that receptors for NKB and Dyn A were present in anterior pituitary cells (17, 35) and, consequently, may be involved in the synthesis and secretion of gonadotropins. Mijiddorj *et al*. (17) reported the presence of NK3R and KOR in gonadotroph cells of the LβT2 line; however, neither NKB nor Dyn activated the FSHβ and LHβ promoter. To the best of our knowledge, there is no information about the pituitary relationship between KNDy peptides and gonadotropins during puberty in ewes. The available reports on the influence of NKB and Dyn A on LH and FSH secretion by pituitary gland cells *in vitro* are ambiguous and do not relate to sheep. We have demonstrated for the first time the direct effect of KiSS-10, NKB and Dyn A on gonadotropin secretion from anterior pituitary cells isolated from pubescent ewes.

## Material and Methods

The protocol of the experimental design and all procedures were approved by the Local Ethics Committee for Animal Experimentation in Lublin (No. 65/2023). The cell culture was prepared using pituitary glands isolated from 10-month-old ewe lambs of the Polish Lowland sheep Uhruska variety (n = 6), housed at the Professor T. Efner Small Ruminant Research Station in Bezek (Poland). The ewes were humanely euthanised by electric shock and exsanguinated at a local slaughterhouse in accordance with applicable regulations. The lambs’ pituitary glands were dissected and transported within 1 h to the laboratory in cold Dulbecco’s modified Eagle’s medium (DMEM) (about 3–5°C) supplemented with 0.08% glucose, 0.59% 4-(2-hydroxyethyl)-1-piperazineethanesulfonic acid (HEPES), 0.1% bovine serum albumin, and gentamicin (20 μg/mL). The anterior and posterior lobes of the pituitary were separated by blunt dissection. The anterior pituitary tissue was minced and repeatedly digested with 0.25% trypsin (10 min, 37°C). After each digestion run, the cells were washed three times in DMEM and centrifuged (1,200 rpm, 10 min). After the last centrifugation, the pituitary cells were passed through a 60-μm nylon filter and counted in a Bürker’s chamber. Cell viability evaluated using the 0.4% trypan blue dye exclusion test was higher than 96%. The pituitary cells (250,000 cells/mL) were then resuspended in McCoy’s 5A medium containing 2.5% foetal calf serum, 10% horse serum, 0.59% HEPES, a mixture of amino acids and vitamins, and gentamicin (20 μg/mL) (adjusted to pH 7.4), and seeded into 24-well culture plates (1 mL/well). The cells were allowed to attach for 96 h at 37°C under a 5% CO_2_ atmosphere (4, 7, 25, 26, 31) until the start of the experiments. During this period, the viability of both the cells suspended in the medium and those already attached to the bottom of the well was also assessed using the 0.4% trypan blue dye exclusion test. Cells that had adhered were detached from the bottom of selected wells using trypsin to determine their viability. The cell viability was estimated at 97% after 24 and 48 h and 96% after 72 and 96 h. After cells’ attachment to the dishes and formation of a monolayer, they were incubated in McCoy’s 5A medium without neuropeptides (which served as the control) or with 10^−11^, 10^−10^, 10^−9^, 10^−8^ and 10^−7^ M of KiSS-10, NKB or Dyn A. After 4, 12 and 24 h of the experiment, the media were collected and stored at −20°C to determine the cumulative concentration of LH and FSH by ELISA using species-specific antibodies (Sheep LH ELISA Kit or FSH ELISA Kit; Sunred Biological Technology, Shanghai, China). The intra- and inter-assay coefficients of variations of the assay for LH and FSH were <10% and <12%, respectively. The LH and FSH secretion levels were expressed as the concentration (mIU/mL) of the hormone released into the culture medium by 250,000 cells within 4–24 h.

### Statistical analysis

The results were calculated using Statistica 13.0 PL (TIBCO, Palo Alto, CA, USA) and expressed as a mean and standard deviation (x ± SD). Differences with a P-value ≤ 0.05 were considered significant. Pearson linear correlation coefficients were calculated to assess the relationships between the analysed variables, which were KiSS-10 concentration and LH or FSH secretion, NKB concentration and LH or FSH secretion and Dyn A concentration and LH or FSH secretion.

## Results

### Influence of KiSS-10 (10^−11^–10^−7^ M) on LH secretion from ovine pituitary cells *in vitro*

**Fig. 2.**
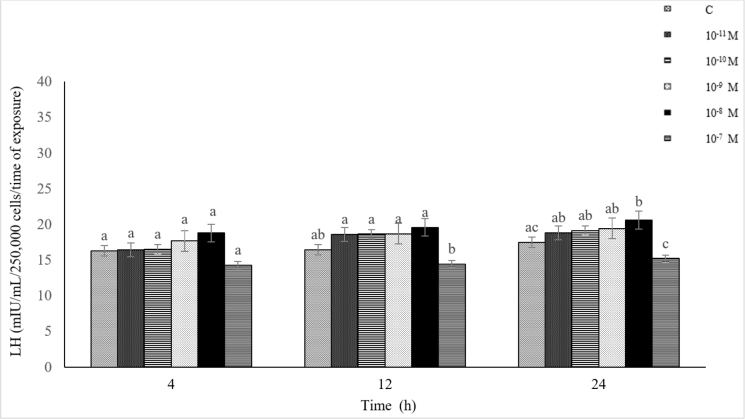
Influence of kisspeptin-10 (10^−11^–10^−7^ M) on luteinising hormone (LH) secretion from ovine pituitary cells *in vitro* a, b, c – mean values obtained at a specific incubation time marked with different letters differ statistically significantly (P-value ≤ 0.05)

The effect of KiSS-10 on LH secretion was dependent on the time of exposure and its concentration in the culture medium (10^−11^–10^−7^ M). Kisspeptin-10 at 10^−11^–10^−8^ M caused an increase in the LH secretion compared to the control throughout the experiment. The highest LH secretion was recorded after the exposure of the cells to 10^−8^ M of KiSS-10, with a maximum after 24 h. This value was statistically significantly higher (P-value ≤ 0.05) than in the control. However, at the highest concentration used (10^−7^ M), KiSS-10 reduced gonadotropin release throughout the entire experiment compared to the control and cultures treated with it at the lower concentrations. Statistically significantly higher (P-value ≤ 0.05) secretion of LH was induced by KiSS-10 applied at the concentrations of 10^−11^–10^−8^ M compared to 10^−7^ M KiSS-10 after 12 and 24 h of incubation. The results revealed a high or very high negative correlation between the concentration of KiSS-10 (10^−11^–10^−7^ M) and the LH secretion from the pituitary cells (r = −0.76, r = −0.96 and r = −0.91 after 4, 12 and 24 h, respectively). However, a positive correlation was found between the KiSS-10 concentrations and the LH secretion only when the neuropeptide was in the concentration range of 10^−11^–10^−8^ M (r = 0.90, r = 0.99 and r = 0.96 after 4, 12 and 24 h, respectively) (Fig. 2).

### Influence of KiSS-10 (10^−11^–10^−7^ M) on FSH secretion from ovine pituitary cells *in vitro*

**Fig. 3.**
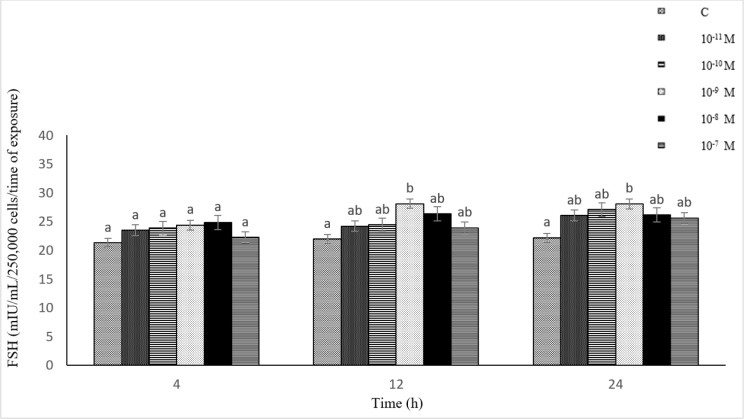
Influence of kisspeptin-10 (10^−11^–10^−7^ M) on follicle-stimulating hormone (FSH) secretion from ovine pituitary cells *in vitro* a, b – mean values obtained at a specific incubation time marked with different letters differ statistically significantly (P-value ≤ 0.05)

Kisspeptin used at all the concentrations caused an increase in FSH secretion compared to the control throughout the experiment. A statistically significant (P-value ≤ 0.05) increase in FSH secretion was observed after 12 and 24 h at the 10^−9^ M concentration compared to the control. However, the exposure of the cells to 10^−7^ M of kisspeptin in the culture medium caused a slight decrease in FSH secretion throughout the experiment compared to secretion in cultures with KiSS-10 applied at the lower concentrations. No statistically significant differences (P-value ≥ 0.05) were found between the FSH secretion levels under the influence of the different KiSS-10 concentrations (10^−11^–10^−7^ M) at any specific incubation time. The results showed a negative correlation between the concentration of KiSS-10 (10^−11^–10^−7^ M) and FSH secretion from the pituitary cells (r = −0.81, r = −0.43 and r = −0.59 after 4, 12 and 24 h, respectively) (Fig. 3).

### Influence of NKB (10^−11^-10^−7^ M) on LH secretion from ovine pituitary cells *in vitro*

**Fig. 4.**
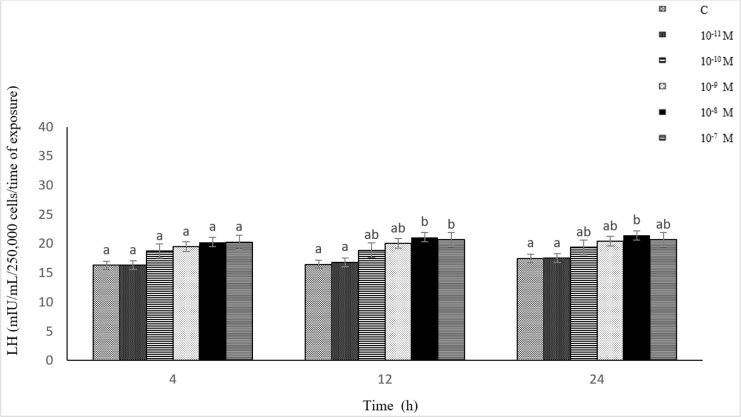
Influence of neurokinin B (10^−11^–10^−7^ M) on luteinising hormone (LH) secretion from ovine pituitary cells *in vitro* a, b – mean values obtained at a specific incubation time marked with different letters differ statistically significantly (P-value ≤ 0.05)

The effect of NKB on LH secretion, similarly to the effect of kisspeptin, was dependent on its concentration in the culture medium and the duration of the experiment. The exposure of the cells to all the NKB doses used had a stimulating effect on LH secretion relative to the control. A marked increase in secretion was induced by 10^−10^–10^−7^ M of NKB after 4–24 h. The strongest stimulatory action (P-value ≤ 0.05) was observed after the 12- and 24-h treatments with 10^−8^ M of NKB. Statistically significantly higher (P-value ≤ 0.05) secretion of LH under the influence of NKB was found at the concentration of 10^−8^–10^−7^ M (after 12 h) and 10^−8^ M (after 24 h), compared to the concentration of 10^−11^ M. The results showed a low positive correlation between the concentration of NKB (10^−11^–10^−7^ M) and LH secretion from the pituitary cells (r = 0.48, r = 0.45, and r = 0.37 after 4, 12 and 24 h, respectively) (Fig. 4).

### Influence of NKB (10^−11^–10^−7^ M) on FSH secretion from ovine pituitary cells *in vitro*

**Fig. 5.**
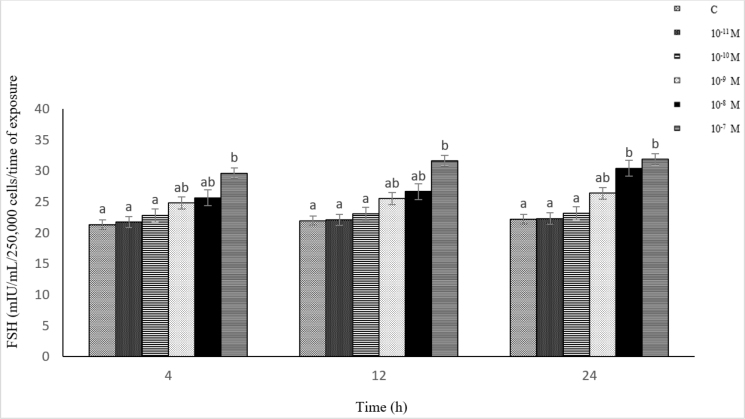
Influence of neurokinin B (10^−11^–10^−7^ M) on follicle-stimulating hormone (FSH) secretion from ovine pituitary cells *in vitro* a, b – mean values obtained at a specific incubation time marked with different letters differ statistically significantly (P-value ≤ 0.05)

The exposure of the cells to 10^−11^–10^−7^ M of NKB resulted in elevated FSH secretion throughout the experiment compared to the control. The most pronounced stimulating effect on FSH secretion was found after 4, 12 and 24 h at 10^−7^ M of NKB. Follicle-stimulating hormone secretion reached a maximum after the 12- and 24-h exposure of the cells to the highest concentration used (10^−7^ M). This value was statistically significantly higher (P-value ≤ 0.05) than in the control and cultures with NKB at the lower concentrations. Statistically significantly higher (P-value ≤ 0.05) secretion of FSH was induced by NKB at the concentration of 10^−7^ M (after 4 and 12 h) and 10^−8^–10^−7^ M (after 24 h) than by this neuropeptide at 10^−11^–10^−10^ M. The results revealed a high positive correlation between the concentration of NKB (10^−11^–10^−7^ M) and FSH secretion from the pituitary cells (r = 0.89, r = 0.90 and r = 0.73 after 4, 12 and 24 h, respectively) (Fig. 5).

### Influence of Dyn A (10^−11^–10^−7^ M) on LH secretion from ovine pituitary cells *in vitro*

**Fig. 6.**
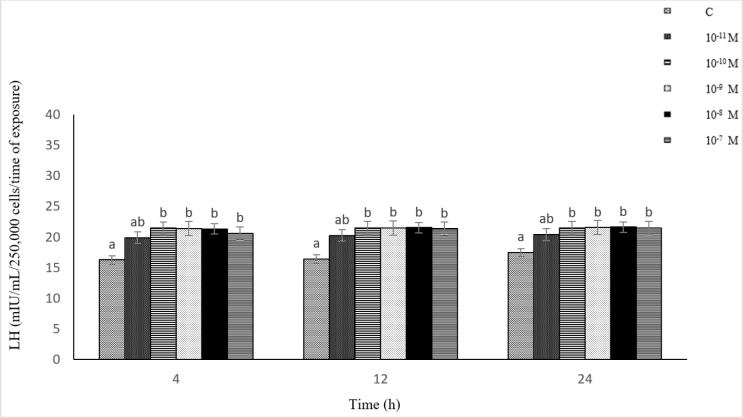
Influence of dynorphin A (10^−11^–10^−7^ M) on luteinising hormone (LH) secretion from ovine pituitary cells *in vitro* a, b – mean values obtained at a specific incubation time marked with different letters differ statistically significantly (P-value ≤ 0.05)

The influence of Dyn A on LH secretion was dependent on the time of exposure and its concentration in the culture medium. The exposure of the cells to 10^−10^–10^−7^ M of Dyn A caused a statistically significant (P-value ≤ 0.05) increase in LH secretion compared to the control. Luteinising hormone secretion reached its maximum level after 24 h in response to 10^−8^ M of Dyn A. However, no statistically significant differences were found between the LH secretion levels under the influence of the different Dyn A concentrations (10^−11^–10^−7^ M) at a specific incubation time. There was no statistically significant correlation between the Dyn A (10^−11^–10^−7^ M) concentration and LH secretion (r = −0.24, r = 0.17 and r = 0.26 after 4, 12 and 24 h, respectively) (Fig. 6).

### Influence of Dyn A (10^−11^–10^−7^ M) on FSH secretion from ovine pituitary cells *in vitro*

The exposure of the cells to Dyn A resulted in an increase in FSH secretion at all the doses used throughout the experiment compared to the control. A significant effect (P-value ≤ 0.05) was observed at the concentrations of 10^−11^–10^−9^ M after 4–24 h. The strongest stimulatory action (P-value ≤ 0.05) was observed after 24 h at 10^−11^ M of Dyn A.

**Fig. 7.**
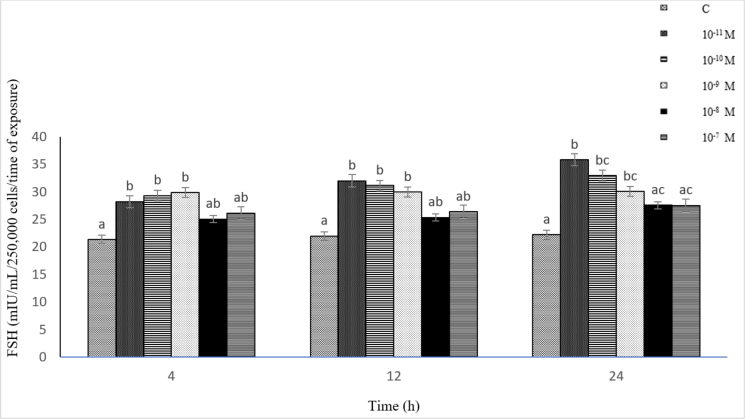
Influence of dynorphin A (10^−11^–10^−7^ M) on follicle-stimulating hormone (FSH) secretion from ovine pituitary cells *in vitro* a, b, c – mean values obtained at a specific incubation time marked with different letters differ statistically significantly (P-value ≤ 0.05)

Statistically significantly higher (P-value ≤ 0.05) secretion of FSH was induced by Dyn A at the concentration of 10^−11^ M compared to its effect at 10^−8^–10^−7^ M after 24 h. The results indicated a moderate negative relationship between the concentration of Dyn A (10^−11^–10^−7^ M) and FSH secretion from the pituitary cells (r = −0.50, r = −0.57 and r = −0.57 after 4, 12 and 24 h, respectively) (Fig. 7).

## Discussion

It is known that three neuropeptides (KiSS-10, NKB and Dyn A) produced by the KNDy subpopulation in the ARC region of the hypothalamus can regulate reproductive functions during sexual development. Detailed data of their characteristics will help to elucidate the pathomechanism of delayed puberty or other neuroendocrine disorders leading to infertility (2, 3, 11, 22, 24, 33). The evidence that KiSS-1 causes an increase in gonadotropin secretion in a GnRH-dependent manner was provided by Navarro *et al*. (21), who indicated that the central administration of KiSS-1 to immature female rats enhanced their plasma LH levels. Moreover, in studies conducted on sheep, Messager *et al*. (16) reported that intraventricular administration of kisspeptin caused a rapid increase in LH secretion during the first 2 h of the experiment in 2- to 3-year-old Ile de France ewes during the anoestrous season. It was also shown that a single intravenous injection of 0.5, 1 or 2 mg of KiSS-10 notably increased LH secretion in three-month-old Small-tail Han female lambs within 15 min (36). In addition, an *in vitro* experiment conducted by Kosior-Korzecka *et al*. (8) indicated that KiSS-10 applied at 10^−11^–10^−9^ M after 48 h caused a high increase in GnRH, which induced FSH secretion by anterior pituitary cells isolated from six-month-old Polish Lowland ram lambs. Furthermore, the expression of GPR54 was detected in the anterior pituitary gland (14, 29); therefore, KiSSs can act directly on pituitary cells (8, 26, 27). As reported by Radwańska and Kosior-Korzecka (26), after 2 h KiSS-10 at 10^−11^–10^−8^ M increased thyroid-stimulating hormone (TSH) secretion *in vitro* from pituitary cells isolated from six-month-old ewe lambs of the SCP line (50% Suffolk + 25% Romanov + 25% Polish Lowland Sheep). These authors also demonstrated that TSH secretion reached a maximum level after 2 h of the action of KiSS-10 at the 10^−11^ M concentration. In turn, our study shows that KiSS-10 also had an impact on gonadotropin secretion by anterior pituitary cells isolated from pubescent ewes. The ewes, predisposed to delayed puberty, were multiple offspring of high-body weight mothers and did not ovulate until their tenth month (27). Since there are no studies in this field carried out on sexually mature sheep and those predisposed to delayed puberty, the present *in vitro* experiment was conducted on anterior pituitary cells isolated from such, and namely these 10-month-old ewes of the Polish Lowland sheep, Uhruska variety. In our research, we demonstrated an increase in the LH and FSH secretion after 4–24 h exposure to 10−^11^–10^−8^ M and 10^−11^–10^−7^ M of KiSS-10, respectively, throughout the experiment. A statistically significant (P-value ≤ 0.05) increase in the LH secretion was recorded after the exposure of the cells to 10^−8^ M of KiSS-10, with a maximum after 24 h of incubation; in turn, the highest FSH secretion was observed after 12 and 24 h at 10^−9^ M of kisspeptin compared to the control. Drawing a comparison with the effect of KiSS-10 in other animal species, we can state that our findings are consistent with the report by Suzuki *et al*. (30), which indicated that KiSS-10 stimulated LH secretion from porcine anterior pituitary cells at the doses of 10^−7^ M and 10^−6^ M and from bovine anterior pituitary cells at 10^−6^ M and 10^−5^ M. Based on our previous findings (9), KiSS-10 also caused significant enhancement of LH secretion by porcine pituitary cells *in vitro* compared to the control, especially after 30-h exposure of the cells to 10^−8^ M of kisspeptin. These data also confirm that KiSS-10 can directly affect gonadotropic cells.

Single reports indicated that NKB and Dyn A may also be directly involved in the synthesis and/or secretion of hormones at the anterior pituitary level. Mun *et al*. (19) observed that the expression of gonadotropic hormone in female Nile tilapia was increased after treatment of pituitary cell cultures with the NKB peptide, especially at concentrations of 10^−8^ M and 10^−6^ M. Based on data reported by Mizrahi *et al*. (18), the intraperitoneal injection of an NKB analogue caused an increase in FSH and LH secretion in sexually mature female Nile tilapia after 1 h of the procedure. Mizrahi *et al*. also demonstrated that neurokinin B and its analogue enhanced the mRNA expression of FSHβ and LHβ (18). In addition, Mijiddorj *et al*. (17) found a receptor of NKB and Dyn in gonadotroph LβT2 cells (a mouse pituitary cell line) and the somatolactotroph GH3 line (rat pituitary cells). However, to the best of our knowledge, there are no such data on sheep. Therefore, in the present study, we investigated any effect of NKB or Dyn A on gonadotropin secretion from anterior pituitary cells isolated from pubescent ewes. Our results showed that the exposure to 10^−11^–10^−7^ M of NKB resulted in elevated LH and FSH secretion by ovine pituitary cells *in vitro* throughout the experiment. The LH secretion reached a maximum after the 24-h treatment with 10^−8^ M of NKB. In turn, the highest level of FSH secretion was observed after the 24-h exposure of the cells to the concentration of 10^−7^ M. We also observed that Dyn A applied at the 10^−11^–10^−7^ M concentration caused a statistically significant (P-value ≤ 0.05) increase in the LH secretion compared to the control. The LH secretion reached the highest level after 24 h in response to 10^−8^ M. In addition, the Dyn A neuropeptide caused the highest increase in the LH secretion throughout the experiment. Furthermore, the exposure of the cells to Dyn A also resulted in an increase in the FSH secretion at all the concentrations used throughout the experiment compared to the control, its secretion reaching its maximum level when acted on by 10^−11^ M of Dyn A for 24 h. This value was significantly higher (P-value ≤ 0.05) than in the control. To summarise the effects of the three neuropeptides on gonadotropin secretion: the most significant stimulating effect on LH and FSH secretion by the pituitary cells of pubescent ewes compared to the control was exerted by Dyn A, and the most pronounced dose-dependent effect of the neuropeptides on FSH secretion was observed during the action of NKB throughout the experiment. Unfortunately, it is difficult to compare the present results, because to the best of our knowledge there have been no similar studies on higher vertebrates in this field.

## Conclusion

In this study, we characterised the ability of NKB, KiSS-10 and Dyn A to stimulate LH and FSH secretion from pituitary cells isolated from pubescent ewe lambs. The results of our study further document the novel role of NKB, KiSS-10 and Dyn A in the neuroendocrine regulation of gonadotropin secretion at the pituitary level. The function of these neuropeptides may consist in the initiation of reproductive activity, which leads to the occurrence of the first ovulation and achievement of sexual maturity. A detailed explanation of the role of KiSS-10, NKB and Dyn A in gonadotropin secretion by the anterior pituitary gland in sheep and the determination of their impact on the regulation of the HPO axis during sexual maturation or in the pathomechanism of delayed puberty require further research.
